# Empowering Caregiver Well-Being With the Adhera Caring Digital Program for Family Caregivers of Children Living With Type 1 Diabetes: Mixed Methods Feasibility Study

**DOI:** 10.2196/66914

**Published:** 2025-07-09

**Authors:** Antonio de Arriba Muñoz, Elisa Civitani Monzon, Maria Pilar Ferrer, Marta Ferrer-Lozano, Silvia Quer-Palomas, Joia Nuñez, Alba Xifra-Porxas, Francesca Aimée Mees Mlatiati, Ioannis Bilionis, Ricardo C Berrios, Luis Fernández-Luque

**Affiliations:** 1 Pediatric Endocrinology Unit, Miguel Servet Children's University Hospital, Zaragoza, Spain; 2 Adhera Health Inc, 101 Cooper St., Santa Cruz, CA, 95060, United States, 1 831 345 5357

**Keywords:** type 1 diabetes, caregiver wellbeing, digital health, digital program, coaching, diabetic, diabetes, T1D, pediatrics, children, chronic, endocrinology, CGM, glucose, caregiving, caregiver, carer, informal care, family care, parents, parental, guardian, continuous glucose monitoring

## Abstract

**Background:**

Caregivers of children living with type 1 diabetes (T1D) face multiple challenges that significantly impact their mental health and quality of life. The well-being of caregivers directly affects the management of the child’s condition. The Adhera Caring Digital Program (ACDP) is a comprehensive, digitally delivered program, designed to support family caregivers in enhancing self-management and well-being. This study aims to assess how the ACDP influences caregivers’ mood, emotional well-being, and health-related quality of life within the context of T1D.

**Objective:**

This study aimed to evaluate the impact of ACDP on caregivers’ psychological well-being and caregiving outcomes.

**Methods:**

This was a two-step, prospective, mixed methods study targeting caregivers of children living with T1D who were under the care of a pediatric endocrinologist at Miguel Servet Children’s University Hospital in Zaragoza, Spain. In substudy 1 (SS1), qualitative and quantitative data were collected to optimize the ACDP. In substudy 2 (SS2), caregivers used the optimized ACDP for three months. Psychometric assessments were conducted at baseline and follow-up to evaluate positive mood states, general well-being, self-efficacy, and lifestyle behaviors. This paper focuses on SS2.

**Results:**

Ninety caregivers participated in SS2. Positive affect significantly increased (*P*<.001), and negative affect decreased (*P*<.001) on the Positive and Negative Affect Schedule (PANAS). Depression, anxiety, and stress scores were reduced (*P*<.001) on the Depression, Anxiety and Stress Scale-21 Items (DASS-21). General well-being, measured by the Mental Health Continuum-Short Form (MHC-SF) and self-efficacy, assessed using General Self-Efficacy Scale (GSE), improved significantly (*P*<.001). Health-related quality of life (HrQoL) scores and Mediterranean Diet Quality Index scores increased modestly (*P*=.03, and *P*=.04, respectively).

**Conclusions:**

The ACDP intervention improved caregivers’ psychological well-being and self-efficacy. These findings highlight the potential of digital solutions to support caregiver mental health and positively influence diabetes management. Future research should explore long-term outcomes and scalability.

## Introduction

Children living with type 1 diabetes (T1D) face unique challenges, including deficient insulin production, psychosocial stress, stigmatization, social isolation, and bullying, which can negatively impact their quality of life [1,2]. Caregivers’ are often the primary source of support for these children, and experience significant emotional burdens that are closely linked to the children’s health–related quality of life (HrQoL) [3-7]. Therefore, addressing caregiver well-being is critical for holistic diabetes management [6].

Comprehensive strategies and programs that prioritize caregiver emotional health have shown promise in improving outcomes for both caregivers and children [8,9]. For instance, programs such as PRISM-P (Promoting Resilience in Stress Management for Parents) have demonstrated the efficacy of resilience-building interventions for parents of children living with serious illnesses [9]. Positive mood states in caregivers are critical predictors of HrQoL in adolescents with T1D [10]. The diagnosis of a chronic condition such as T1D in a child is often stressful and potentially traumatic [11]. Furthermore, scalable digital interventions such as the Remedy to Diabetes Distress (R2D2) program underscore the growing need for innovative, technology-enabled solutions to address caregiver distress [12].

Achieving optimal HrQoL for children living with T1D may also require providing caregivers with personalized emotional and behavioral support [13]. This issue should be examined from the perspectives of mental health and technology acceptance theoretical frameworks [14]. The psychological well-being of caregivers is closely tied to the HRQoL and treatment adherence of children living with T1D [15]. For example, the PsyVoice study highlights the importance of understanding expectations surrounding voice-based digital health solutions to manage diabetes distress among children and their caregivers [16]. Furthermore, a recent narrative synthesis of systematic reviews underscores ongoing developments in the design and delivery of self-management support for children and young people with diabetes, emphasizing the necessity of tailored interventions to meet diverse needs [17].

The Adhera Caring Digital Program ACDP is a comprehensive, digitally delivered program designed to support the physical and mental well-being of caregivers of children living with chronic conditions. By providing personalized emotional and behavioral support, the ACDP seeks to improve self-management, mental health. and overall well-being for both patients and their families. This study evaluates the impact of the ACDP on caregivers’ positive mood states, emotional health, and perceived HrQoL in the context of T1D.

## Methods

### Design and Setting

This study used a two-step, prospective, mixed methods (qualitative-quantitative) feasibility trial, composed of two substudies. Substudy (SS1) gathered qualitative and quantitative data to inform the optimization of the ACDP. Substudy 2 (SS2) evaluated the optimized intervention over a three-month period. Caregivers of children living with T1D under the care of a pediatric endocrinologist at the Miguel Servet Children’s University Hospital (Zaragoza, Spain), following general clinical Spanish practices, participated in this study.

### Sample Size and Eligibility Criteria

One hundred caregivers were recruited for SS2. Inclusion criteria were (1) caregivers who are legal guardians of children living with T1D under 18 years of age; (2) child’s T1D diagnosis for at least three months; (3) use of continuous glucose monitor; (4) caregivers’ willingness to use the mobile solution and share data.

Exclusion criteria included only one legal guardian per child could participate, prior participation in SS1, and incomplete or refusal to provide consent.

### Digital Solution

The ACDP supports the physical and mental well-being of family caregivers of individuals with chronic conditions, enhancing self-management and health outcomes. This noninvasive, digitally delivered program offers condition-specific educational content, personalized motivational messages, and self-management tools, using data from wearables and patient-reported outcomes. It includes Adhera Collaboration, a health coaching service, and leverages the Adhera Health AI-driven Health Recommender System for personalized interventions. ACDP is part of the Adhera Health Precision Digital Companion Platform, developed according to best practices in data protection and quality management, following ISO 27001 and ISO 13465 guidelines. The family caregivers accessed the digital program via a mobile application which is compatible with both Android and Apple operative systems. Screenshots of the ACDP can be found in the Multimedia Appendix 1.

The ACDP was applied to families managing T1D due to the significant caregiving demands associated with this condition. The program was tailored to address the psychological and emotional challenges faced by caregivers in supporting children with T1D, including continuous glucose monitoring, insulin management, and responding to hypoglycemic and hyperglycemic events. These challenges made T1D an optimal use case for evaluating the intervention’s impact on caregiver well-being.

### Study Procedures

SS1 aimed to identify caregivers’ psychological burdens and perceived barriers and facilitators associated with adopting the ACDP. Participants used the digital intervention for one month, and their feedback was used to generate an optimized version of the platform. Full details of SS1 can be found in Multimedia Appendix 1.

SS2 assessed the impact of the optimized ACDP on caregivers’ psychological well-being, mood and HrQoL. Demographic data collected included caregiver gender, age, marital status, and education level, as well as the children’s gender, age, and time since T1D diagnosis. These characteristics were analyzed to understand the context and diversity of the caregiving population. Furthermore, psychometric assessments used in this study included the following validated surveys:

- Positive and Negative Affect Schedule (PANAS**)** measures positive and negative affect. Higher scores on the positive affect subscale indicate greater positive emotions, while higher scores on the negative affect subscale indicate greater negative emotions.
- Depression, Anxiety, and Stress Scale (DASS-21) evaluates emotional distress across three domains, including depression, anxiety, and stress. Higher scores indicate greater levels of distress in each domain.
- Mental Health Continuum-Short Form (MHC-SF) assesses general well-being across emotional, psychological, and social domains. Higher scores reflect better overall mental well-being.
- General Self-Efficacy Scale (GSE**)** measures confidence in managing challenging situations. Higher scores indicate greater self-efficacy.
- Clarke Questionnaire typically applied to patients with diabetes to assess hypoglycemia awareness; in this study. it was adapted to capture caregivers’ awareness and understanding of hypoglycemic episodes in their children. Higher scores suggest better awareness and understanding of hypoglycemia.
- KIDSCREEN-10 measures HRQoL in children. Higher scores indicate better HRQoL.
- Mediterranean Diet Quality Index (KIDMED**)** assesses adherence to a Mediterranean diet. Higher scores reflect greater adherence to a Mediterranean dietary pattern.

While KIDSCREEN-10 and KIDMED are primarily validated for ages 8‐18, caregivers completed them to provide insight into their perception of the child’s quality of life and dietary habits, respectively. Their inclusion aimed to explore indirect caregiver influences on child health behaviors.

### Intervention and Measures

The recruitment for SS2 took two months, and participants joined ACDP for three months. Caregivers were onboarded to the Adhera platform through an initial in-person training session at the study site. This session included a demonstration of platform functionality, account setup, and navigation support. Data were collected through the Adhera platform’s integrated survey tools, which allowed participants to complete assessments digitally. Additionally, demographic and baseline data were gathered via paper forms during the initial study visit. At baseline and at follow-up, the data collected included (1) demographic data; (2) multiple daily injections and continuous subcutaneous insulin infusion questionnaire (insulin delivery methods); (3) positive mood by PANAS, (4) distress by DASS-21, general wellbeing by MHC-SF, self-efficacy by GSE; (5) lifestyle questionnaires by KIDMED; (6) hypoglycemia awareness by Clarke questionnaire; (7) Quality of life (HrQoL) by KIDSCREEN-10; (8) system usability scale (SUS; only at 3 months), and (9) continuous glucose monitoring.

### Data Management and Quality Control

All the data gathered in the study was recorded in MicroSoft Forms at Adhera Health servers. Data were processed, evaluated, and stored in an anonymous form following the General Data Protection Regulation (GDPR) regulations. Adhera Health was responsible for data processing in accordance with its data management and quality procedures. Data quality and integrity were ensured through ISO 27001, which is related to the quality management system for ensuring information security.

### Statistical Analysis

Baseline demographic and clinical characteristics of the study cohort were summarized using means (SD) for continuous variables, and frequencies with percentages for categorical variables. Mean values (SD) were presented for each psychometric outcome (eg, stress, quality of life, depressive symptoms) were calculated at baseline (preintervention) and after the 3-month intervention (postintervention). Differences in pre- and postintervention questionnaire scores were assessed using a parametric paired *t* test or a nonparametric Wilcoxon signed-rank test, as appropriate, depending on whether the normality and homogeneity assumptions of the *t* test were met. Effect sizes (η^2^) were calculated to gauge the magnitude of any significant differences. All hypothesis tests were two-sided, and statistical significance was defined as *P*<.05. The statistical analysis was conducted using the Python packages *scipy* (version 1.14.1) and *pingouin* (version 0.5.5).

### Ethical Considerations

All procedures performed in this study were in accordance with the ethical standards of the 1964 Declaration of Helsinki and its later amendments or comparable ethical standards. The study posed minimal risk to the participants. The anonymized information collected was handled following the right of privacy and anonymity according to the rules of GDPR (EU Regulation 2016/679). Prior to the commencement of the study at Miguel Servet University Hospital, the protocol and its associated documents (information sheet for the patient and informed consent form) were submitted to the CEICA (Aragonese Ethical Committee for Clinical Research). A written favorable opinion or approval was obtained from CEICA. Written informed consent to participate in the study was obtained from all patients before any study-related activities were carried out. Moreover, to comply with local regulations, for children 12 years and older, additional consent was obtained. Data were anonymized as per GDPR standards. The study participants were not compensated monetarily. However, they were provided with free access to the digital health platform for the duration of the study, which included tailored support and resources.

## Results

### Demographics

**Figure 1.**
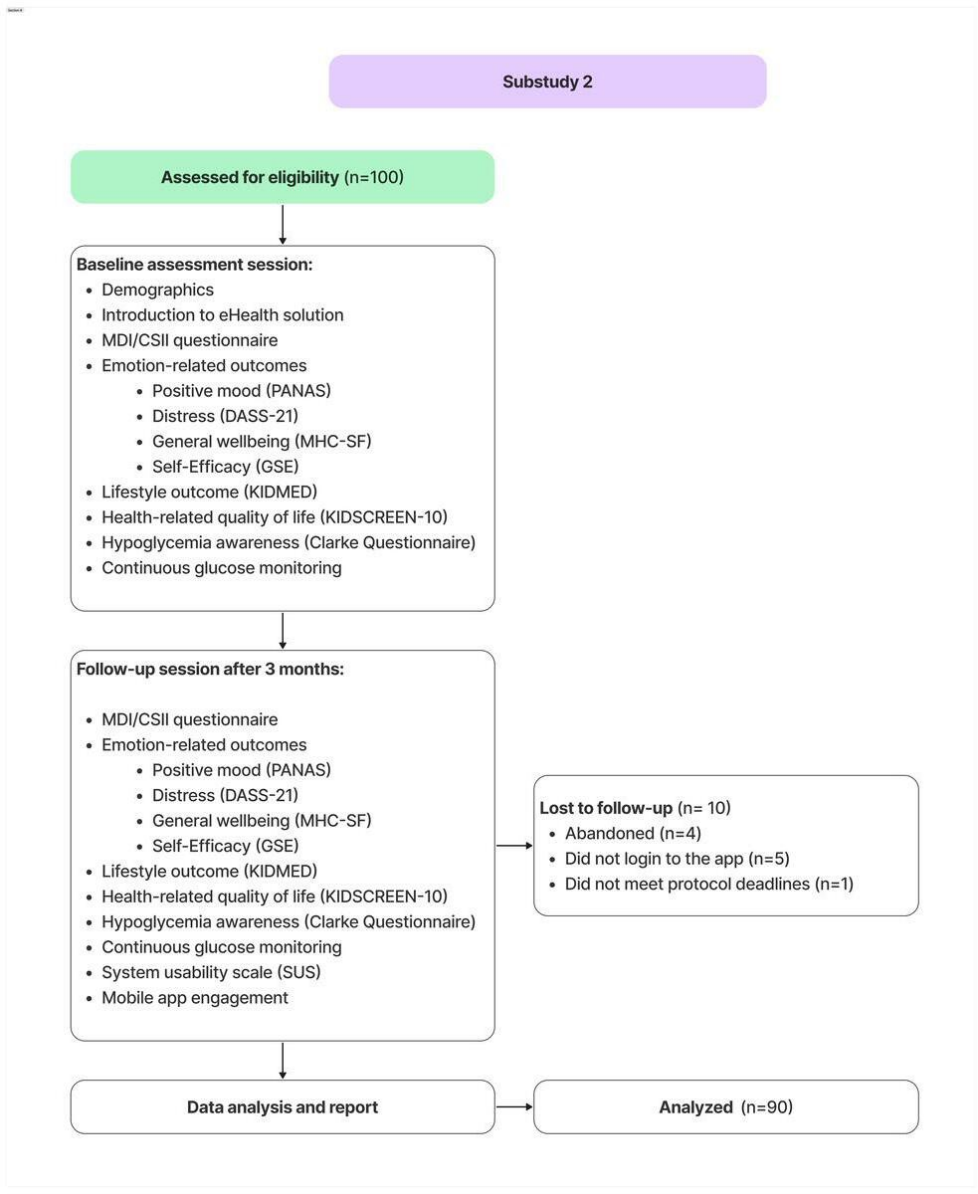
Substudy 2 diagram. DASS-21:depression, anxiety and stress scale-21 items; GSE: general self-efficacy scale; MDI/CSII: multiple daily injections and continuous subcutaneous insulin infusion; MHC-SF: mental health continuum-short form; PANAS: positive and negative affect schedule; SUS: system usability scale.

Of the 100 caregivers recruited, 90 completed the study (Figure 1). Reasons for drop out were as follows: 4 caregivers abandoned the study, 5 did not log in to the application, and 1 did not meet the protocol deadlines. Participants’ mean age was 45.15 (SD 6.03) years, and they were predominantly female (71/90, 78.9%). The mean age of the children was 10.78 (SD 3.34) years, with a mean time since diagnosis of 4.42 (SD 3.38) years. Full demographic details are provided in Table 1. The differences in pre- and postintervention questionnaire are presented in Table 2.

**Table 1.** Demographics of participants of substudy 2.

Characteristics	Participants (N=90), n (%)
Caregiver’s gender, n (%)	
Men	19 (21.1)
Women	71 (78.9)
Caregiver’s age (years), mean (SD)	45.27 (5.04)
Caregiver’s marital status, n (%)	
Single	3 (3.4)
Married	79 (87.8)
Divorced	8 (8.9)
Education, n (%)	
Primary education	2 (2.2)
Secondary education/high school	13 (14.4)
Professional training	35 (38.9)
University degree	40 (44.4)
Child’s gender, n (%)	
Male	51 (56.7)
Female	39 (43.3)
Child’s age (years), mean (SD)	10.78 (3.33)
Time since diagnosis (years), mean (SD)	4.42 (3.38)
Therapy, n (%)	
CSII^a^	46 (51.1)
MDI^b^	44 (48.9)

a CSII: subcutaneous insulin infusion.

b MDI: multiple daily injections.

**Table 2.** Comparison of family caregiver characteristics at baseline and three months after the start of the intervention in Substudy 2.

Variables	At baseline(mean, SD)	At 3 months(mean, SD)	*t* test (*df*)/nonparametric test	*P* value	Effect size(η^2^)^a^
PANAS^b^					
Positive affect	34.26 (7.69)	37.40 (6.95)	-4.7^c^	<.001	.044
Negative affect	24.34 (8.83)	21.23 (8.36)	941.0^d^	<.001	.032
DASS-21^e^					
Depression scale	4.21 (4.65)	2.96 (3.90)	550.5^d^	<.001	.021
Anxiety scale	3.54 (4.24)	2.50 (3.65)	362.5^d^	<.001	.017
Stress scale	8.98 (5.30)	5.75 (4.7)	536.5^d^	<.001	.048
MHC-SF^f^					
General well-being	0.61 (0.19)	0.67 (0.17)	-3.4^c^	.001	.021
Emotional well-being	0.62 (0.21)	0.70 (0.22)	661.0^d^	<.001	.028
Psychological well-being	0.68 (0.21)	0.72 (0.17)	957.5^d^	.02	.011
Social well-being	0.52 (0.22)	0.58 (0.22)	741.5^d^	<.001	.017
GSE^g^	30.44 (5.18)	32.28 (4.81)	785.5^d^	<.001	.033
KIDSCREEN-10 index					
Total Score (T-value)	57.76 (15.22)	60.39 (15.58)	1164.5^d^	.03	.007
KIDMED questionnaire	8.60 (1.80)	9.00 (1.90)	716.0^d^	.04	.011
Knowledge of T1D^h^	7.47 (2.18)	7.59 (1.66)	1092.5^d^	.62	<.001
Clarke hypoglycemia score	2.60 (1.06)	2.48 (1.01)	496.5^d^	.22	.003

a η2>0.01 indicates a significant effect.

b PANAS: positive and negative affect schedule.

c *t* test (89).

d Nonparametric test.

e DASS-21: depression, anxiety and stress scale-21 item.

f MHC-SF: mental health continuum-short form..

g GSE: general self-efficacy scale..

h T1D: type 1 diabetes.

### Pre-Post Intervention Results

Significant improvements were observed across multiple outcomes (see Table 2). Positive affect increased from 34.26 (SD 7.69) to 37.40 (SD 6.95; *P*<.001) on the PANAS. Negative affect decreased from 24.34 (SD 8.83) to 21.23 (SD 8.36; *P*<.001) on the PANAS. Depression, anxiety, and stress scores on the DASS-21 significantly reduced across all domains, including significant decreases in depression (mean difference =1.25, *P*<.001), anxiety (mean difference=1.04; *P*<.001), and stress (mean difference=3.23; *P*<.001).

General well-being, as assessed by the MHC-SF, showed significant improvements across domains such as general well-being (mean change=0.06; *P*=.001), emotional (mean change=0.08; *P*<.001), psychological (mean change = 0.04; *P*=.02), and social well-being (mean change=0.06, *P*<.001). The GSE scores increased from 30.44 (SD 5.18) to 32.28 (SD 4.81; *P*<.001) and the HRQoL scores improved significantly (*P*=.03). The Mediterranean Diet Quality Index scores were also improved (*P*=.04).

## Discussion

### Principal Findings

This feasibility study shows that a 3-month digital intervention can significantly improve the psychological wellbeing and emotional health of caregivers of children living with T1D. The intervention effectively increased positive affect, decreased negative affect, and reduced depression, anxiety, and stress levels among caregivers. These findings align with existing research emphasizing the importance of caregiver support in pediatric chronic condition management [18,19].

The observed improvements in survey scores suggest clinically meaningful benefits. For example, reductions in DASS-21 scores correspond to a shift from moderate to mild emotional distress categories for many participants, reflecting improved mental health. Similarly, increases in PANAS positive affect scores and MHC-SF well-being scores indicate enhanced emotional and psychological resilience. These clinically significant changes underscore the impact of the ACDP on caregiver well-being and its potential to support families managing T1D. Higher self-efficacy is associated with better coping strategies and more effective problem-solving [15,20]. Although improvements in T1D knowledge and hypoglycemia awareness were not statistically significant, the observed trends suggest potential benefits [21]. It is possible that with a larger sample size or a longer intervention period, these trends could reach statistical significance, providing a more comprehensive understanding of the intervention’s impact.

Caregiver burden has been strongly linked to adverse psychological effects, including depression, family dysfunction, and difficulties in glycemic control [7,22]. Addressing these challenges is essential for both caregiver well-being and effective diabetes management in children. The combination of digital coaching, motivational messaging, and tailored interventions in ACDP offers a structured approach to alleviating caregiver distress. Additionally, resilience plays a crucial role in mitigating the negative impact of caregiver burden on quality of life [11]. The improvements in self-efficacy and emotional well-being observed in this study highlight how digital interventions can empower caregivers with better coping mechanisms and stress management strategies.

Moreover, the observed improvements in caregiver psychological well-being and self-efficacy align with prior studies, such as PRISM-P and R2D2, which have demonstrated the efficacy of resilience-building and digital health interventions in supporting families managing chronic conditions [9,12]. For instance, the PANAS and DASS-21 score changes in this study are comparable to those reported in similar interventions, indicating a meaningful reduction in caregiver distress and an increase in positive affect. Furthermore, the MHC-SF improvements reflect better emotional and social functioning, consistent with findings from resilience-focused digital interventions. These results underscore the potential of personalized, tech-enabled support to fill critical gaps in caregiver support, as highlighted by Yu et al [21]. However, this study uniquely integrates personalized modules and psychometric assessments tailored to caregivers of children living with T1D, addressing a distinct population need. The significant improvements observed in this study demonstrate the scalability and real-world applicability of the ACDP for enhancing caregiver and family well-being.

This study has several strengths. First, it leverages a personalized digital intervention tailored specifically for caregivers of children with T1D, addressing an important but often overlooked population. The integration of psychometric assessments and AI-driven personalization ensures that the intervention adapts to individual caregiver needs, enhancing its real-world applicability. Additionally, the study employs a mixed methods approach, combining validated psychometric tools with engagement metrics to provide a comprehensive understanding of both subjective and objective outcomes.

Despite these positive outcomes, the study has limitations. The sample size, although adequate for a feasibility study, limits the generalizability of the findings. Additionally, the intervention’s three-month duration may not capture long-term sustainability of the observed benefits. Potential biases in self-reported data and a lack of a control group further constrain the robustness of conclusions. Future research should focus on exploring the long-term effects of digital interventions and their potential to include other domains of caregiver and child well-being. Additionally, examining the cost-effectiveness of such interventions could provide valuable insights for health care providers and policymakers considering the implementation of digital support programs for caregivers.

However, the study’s findings are promising, indicating that digital interventions can effectively support caregivers’ psychological and emotional well-being. The significant improvements in affect, mental health, self-efficacy, and quality of life highlight the potential of such interventions to enhance the caregiving experience and ultimately benefit the well-being of children living with T1D [21]. While improvements in emotional well-being and self-efficacy may indirectly influence caregiving behaviors, these were not directly targeted or assessed in this study. Future research could explore the potential behavioral impacts of digital interventions such as the ACDP.

### Conclusions

The ACDP has demonstrated significant potential in supporting caregivers of children living with T1D. By improving psychosocial well-being and self-efficacy, the intervention offers a scalable solution for enhancing caregiving experiences and, ultimately, child health outcomes. Further studies are warranted to explore long-term impacts and broader applications.

## Supplementary material

10.2196/66914 Multimedia Appendix 1 Substudy 1 and Adhera Caring Digital Program information and captions.

## Abbreviations

ACDP: Adhera Caring Digital Program
DASS-21: depression, anxiety and stress scale-21 items
GDPR: General Data Protection Regulation
GSE: general self-efficacy scale
HrQoL: health-related quality of life
MHC-SF: mental health continuum-short form
PANAS: positive and negative affect schedule
SS1: Substudy 1
SS2: Substudy 2
T1D: type 1 diabetes

## References

[1] Andrade C do N, Alves C de A (2019). Relationship between bullying and type 1 diabetes mellitus in children and adolescents: a systematic review. J Pediatr (Rio J).

[2] Nieuwesteeg A, Pouwer F, van der Kamp R, van Bakel H, Aanstoot HJ, Hartman E (2012). Quality of life of children with type 1 diabetes: a systematic review. Curr Diabetes Rev.

[3] Alsaigh R, Coyne I (2019). Mothers’ experiences of caring for children receiving growth hormone treatment. J Pediatr Nurs.

[4] Eckshtain D, Ellis DA, Kolmodin K, Naar-King S (2010). The effects of parental depression and parenting practices on depressive symptoms and metabolic control in urban youth with insulin dependent diabetes. J Pediatr Psychol.

[5] Carona C, Silva N, Crespo C, Canavarro MC (2014). Caregiving burden and parent-child quality of life outcomes in neurodevelopmental conditions: the mediating role of behavioral disengagement. J Clin Psychol Med Settings.

[6] Celano M, Bakeman R, Gaytan O, Smith CO, Koci A, Henderson S (2008). Caregiver depressive symptoms and observed family interaction in low-income children with persistent asthma. Fam Process.

[7] Azimi T, Johnson J, Campbell SM, Montesanti S (2024). Caregiver burden among parents of children with type 1 diabetes: A qualitative scoping review. Heliyon.

[8] Raina P, O’Donnell M, Schwellnus H (2004). Caregiving process and caregiver burden: conceptual models to guide research and practice. BMC Pediatr.

[9] Yi-Frazier JP, Fladeboe K, Klein V (2017). Promoting Resilience in Stress Management for Parents (PRISM-P): an intervention for caregivers of youth with serious illness. Fam Syst Health.

[10] Lord JH, Rumburg TM, Jaser SS (2015). Staying positive: positive affect as a predictor of resilience in adolescents with type 1 diabetes. J Pediatr Psychol.

[11] Luo D, Gu W, Bao Y (2021). Resilience outstrips the negative effect of caregiver burden on quality of life among parents of children with type 1 diabetes: an application of Johnson-Neyman Analysis. J Clin Nurs.

[12] Patton SR, Pierce JS, Fox L, Benson M, Mc Donough R, Clements MA (2022). Remedy to Diabetes Distress (R2D2): development protocol for a scalable screen-to-treat program for families of school-age children. Contemp Clin Trials.

[13] López-Bastida J, López-Siguero JP, Oliva-Moreno J (2019). Health-related quality of life in type 1 diabetes mellitus pediatric patients and their caregivers in Spain: an observational cross-sectional study. Curr Med Res Opin.

[14] Mohr DC, Schueller SM, Montague E, Burns MN, Rashidi P (2014). The behavioral intervention technology model: an integrated conceptual and technological framework for eHealth and mHealth interventions. J Med Internet Res.

[15] Thorsteinsson EB, Loi NM, Rayner K (2017). Self-efficacy, relationship satisfaction, and social support: the quality of life of maternal caregivers of children with type 1 diabetes. PeerJ.

[16] Aguirre Vergara F, Fischer A, Seuring T, de Beaufort C, Fagherazzi G, Aguayo GA (2023). Mixed-methods study protocol to identify expectations of people with type 1 diabetes and their caregivers about voice-based digital health solutions to support the management of diabetes distress: the PsyVoice study. BMJ Open.

[17] Litchfield I, Barrett T, Hamilton-Shield JP (2023). Developments in the design and delivery of self-management support for children and young people with diabetes: a narrative synthesis of systematic reviews. Diabet Med.

[18] Whittemore R, Jaser S, Chao A, Jang M, Grey M (2012). Psychological experience of parents of children with type 1 diabetes: a systematic mixed-studies review. Diabetes Educ.

[19] Bassi G, Mancinelli E, Di Riso D, Salcuni S (2020). Parental stress, anxiety and depression symptoms associated with self-efficacy in paediatric type 1 diabetes: a literature review. Int J Environ Res Public Health.

[20] Grover S, Bhadada S, Kate N (2016). Coping and caregiving experience of parents of children and adolescents with type-1 diabetes: An exploratory study. Perspect Clin Res.

[21] Yu J, Wang Y, Wang H (2023). Association between eHealth literacy, diabetic behavior rating, and burden among caregivers of children with type 1 diabetes: cross-sectional survey study. J Pediatr Nurs.

[22] Balcázar-Hernández L, Huerta-Martínez H, Garrido Magaña E, Nishimura-Meguro E, Jiménez Márquez A, Rivera-Hernández A (2022). Burden in primary informal caregivers of children and adolescents with type 1 diabetes: Is it associated with depression, family dysfunction, and glycemic control?. Front Endocrinol (Lausanne).

