# Supplementary material for: Empowering Caregiver Well-Being With the Adhera Caring Digital Program for Family Caregivers of Children Living With Type 1 Diabetes: Mixed Methods Feasibility Study
Source: JMIR Pediatr Parent. 2025 Jul 9;8:e66914. doi: 10.2196/66914 (PMC12266296; doi:10.2196/66914)

## Appendix SS1

***Sample size and eligibility criteria***

For sub-study 1, 20 caregivers were recruited. The participants of the study are chosen in accordance of the following inclusion criteria: a) caregivers who are legal guardians of patients with type 1 diabetes under 18 years of age; b) participant’s children’s type 1 diabetes debut was at least 3 months prior the start of the study; c) Participant’s children’s must use a continuous glucose monitor; d) participants administer insulin treatment to their children; e) must be users of smartphone and willing to install the mobile solution of the study; f) must sign an informed consent; g) must agree on sharing data on continuous glucose monitoring data of their infants.

The exclusion criteria were as follows: only one legal guardian per child could participate, and participants in sub-study 1 were not eligible to participate in sub-study 2.

***Intervention and Measures***

For sub-study 1, the recruitment period was 3 weeks, and participants joined ACDP® for one month. During the first visit, the variables measures were demographics, knowledge of the disease, and Depression, Anxiety and Stress Scale (DASS-21); during the second visit, System Usability Scale (SUS) was administered, and a semi-structured interview was performed.

***Data management and quality control***

All the data gathered in the study was recorded in MicroSoft Forms at Adhera Health servers. Data was processed, evaluated, and stored in an anonymous form following the GDPR regulation. Interviews were recorded and transcribed. Once the transcriptions were entered into the database the recordings were destroyed. Adhera Health is responsible for data processing, in accordance with its data management and quality procedures. Data quality and integrity are ensured through the application of top information quality management standards according to ISO 27001 related to the quality management system for ensuring information security.

Sub-study 1 diagram

**Results**

In SS1, we gathered qualitative and quantitative data to understand the challenges and facilitators experienced by caregivers following a month-long digital intervention. This initial phase informed the optimization of the Adhera Caring Digital Program® (ACDP) for SS2. In SS2, we evaluated the impact of the refined digital intervention on caregivers' positive mood states, distress levels, general wellbeing, self-efficacy, lifestyle behaviors, hypoglycemia awareness, quality of life, and system usability over a three-month period. The following section presents the findings from both sub-studies, detailing the demographic characteristics of the participants and the effects of the digital intervention on the measured outcomes.

***Demographics***

The sample were 20 caregivers (see table 1). The majority of the participants were female (85%) with a mean age of 45.29 years (SD = 22.42). Most caregivers were married (90%), while a smaller proportion were divorced (10%). Educational levels among caregivers show an even split between those with professional training (45%) and those with university degrees (45%), with only 10% having primary education. Regarding the children being cared for, 75% were male and 25% were female, with an average age of 8.45 years (SD = 4.10). The average time since diagnosis for these children was 2.5 years (SD = 2.07).

|  | **Sub-study 1** |
| --- | --- |
| **Characteristic** | **(n=20)** |
| **Caregiver's Gender, n (%)** |  |
| Male | 3 (15%) |
| Female | 17 (85%) |
| **Caregiver's Age, mean (SD)** | 45.29 (22.42) |
| **Caregiver’s Marital Status, n (%)** |  |
| Single | 0 (0%) |
| Married | 18 (90%) |
| Divorced | 2 (10%) |
| **Education, n (%)** |  |
| Primary Education | 2 (10%) |
| Secondary Education/High school | 0 (0%) |
| Professional Training | 9 (45%) |
| University Degree | 9 (45%) |
| **Child's Gender, n (%)** |  |
| Male | 15 (75%) |
| Female | 5 (25%) |
| **Child's Age, mean (SD)** | 8.45 (4.10) |
| **Time since diagnosis (years), mean (SD)** | 2.5 (2.07) |
| **Therapy, n (%)** |  |
| CSII | - |
| MDI | - |

***DASS-21 at baseline SS1***

At baseline, the Depression Anxiety Stress Scales-21 (DASS-21) results for the sample indicate that the majority of participants fell within the normal range for depression (75%), anxiety (65%), and stress (40%). A small proportion experienced mild levels of depression (15%) and anxiety (20%), with only 10% reporting mild stress. No participants had moderate depression, while moderate levels of anxiety and stress were observed in 10% of each sample. Severe stress was more prevalent (30%) compared to severe anxiety and depression, both of which were not present. Extremely severe depression was reported by 10% of participants, whereas extremely severe anxiety and stress were reported by 5% and 10% of participants, respectively.

**Interviews**

The ACDP® received great positive feedback, with participants valuing its emotional support, practical advice, and educational content on diabetes management. The program's continuous accessibility helped caregivers manage stress and anxiety, offering relief and reassurance. Daily guidance, relaxation features, and motivational messages were particularly beneficial, reducing parental stress and guilt, thus indirectly benefiting the child's health. The intuitive navigation and visually appealing design were appreciated, enhancing ease of use. However, participants suggested improvements such as more nuanced response options in questionnaires, interactive and visual aids for children's understanding, practical tools like a carbohydrate calculator, and more detailed educational content. Additional psychological support, motivational content for parents, expanded focus to include extended family, clearer registration instructions, enhanced nature sounds, audio messages, and personalized relaxation resources were also recommended. These enhancements and challenges were considered for sub-study 2.

## Digital Solution: Adhera Caring Digital Program (ACDP)

Adhera Health is pioneering family-focused pediatric chronic care management through precision digital companion solutions, empowering families to lead healthier and happier lives. Our groundbreaking, clinically validated Adhera® programs are designed to enhance mental and physical wellbeing and address health disparities in families facing conditions such as diabetes, obesity, and autoimmune diseases.

**Development process**

The ACDP was built on the Adhera® Health Precision Digital Companion™ platform, leveraging artificial intelligence and data analytics to deliver personalized, context-aware interventions. The development process included:

- **Stakeholder Engagement:** Input was gathered from caregivers, healthcare professionals, and clinical researchers to identify key stressors and unmet needs.
- **Content Design:** Modules were co-developed and validated by pediatric endocrinologists and psychologists, focusing on emotional well-being, stress management, self-efficacy, and lifestyle behavior changes.
- **Technical Validation:** The platform underwent rigorous usability and functionality testing, ensuring compatibility with both Android and iOS devices.

**Program Content and Format**

The ACDP provided a comprehensive suite of resources and tools, including:

- **Health Coach Support**: Get personalized guidance tailored to your family's unique health needs and goals. Stay motivated and accountable with regular check-ins and encouragement from a dedicated health coach, ensuring that everyone in the family stays on track and achieves their health objectives.
- **Content and Activities**: Empower your family with reliable, up-to-date information on pediatric chronic conditions and related health topics. Improve health literacy and make informed decisions with easy-to-understand content, while also learning preventive care and communication strategies to promote long-term wellness for the whole family.
- **Community Connections**: Join a supportive community of like-minded parents and families. Share experiences, gain emotional support, and feel a sense of belonging while exchanging valuable tips and advice that enrich your family's health journey.
- **Localized Resources**: Conveniently find nearby healthcare providers, support groups, and specialized services tailored to your local community and chronic condition, all in one place.
- **Personal Goal Tracking**: Boost motivation and accountability by setting and tracking personalized goals tailored to your family’s unique needs and circumstances. Reach milestones with confidence as our responsible AI-driven Adhera Health Recommender System provides tailored guidance and support, delivering personalized messages to keep you on track and motivated along your family’s health journey.

**Personalized Engagement**

Engagement was robust, with caregivers making an average of 504 interactions with the app during the intervention. The most frequently accessed features were educational content and motivational messages, reflecting their value in supporting caregiver needs. System Usability Scale (SUS) scores indicated high user satisfaction (85.3 ± 13.7).

A minority of users utilized the chat feature, exchanging over 700 messages with coaches. Discussions in the chat predominantly centered on themes such as "caring for the caregiver," "emotional/behavioral management of children," "family communication," "self-esteem," "diabetes management outside the home," and "sleep problems." These interactions provided tailored, real-time support for specific challenges faced by caregivers.

**Access to Broader Platform**

Study participants accessed the ACDP via the broader Adhera Health platform. This included:

- **Wearable Integration:** Data from continuous glucose monitors (CGMs) were seamlessly integrated into the system, enabling real-time insights.
- **Community Resources:** Participants could explore curated articles and videos on caregiver well-being.
- **Data Visualization:** Interactive dashboards summarized progress in mood, stress, and adherence metrics, fostering self-awareness and informed decision-making.
- **Gamification Elements:** Badges and rewards were offered to incentivize sustained engagement.

**Improvements from SS1 to SS2**

Based on insights from SS1, several enhancements were implemented in SS2 to optimize the user experience and engagement with the ACDP. A new user interface (UI) and improved onboarding flow were introduced to streamline navigation and accessibility. Additional educational content and personalized information modules (PIMs) were integrated to provide more tailored guidance. Wearable device synchronization capabilities were expanded, allowing caregivers to seamlessly track relevant health data. A health coaching service via video calls was added to offer real-time support. To further boost engagement, two new interactive features were introduced: a bonus content system unlocked through user interactions and a time-sensitive content mechanism that encouraged daily engagement by making specific materials available for only 24 hours. These refinements aimed to enhance usability, personalization, and sustained participation in the program.

## Screenshots


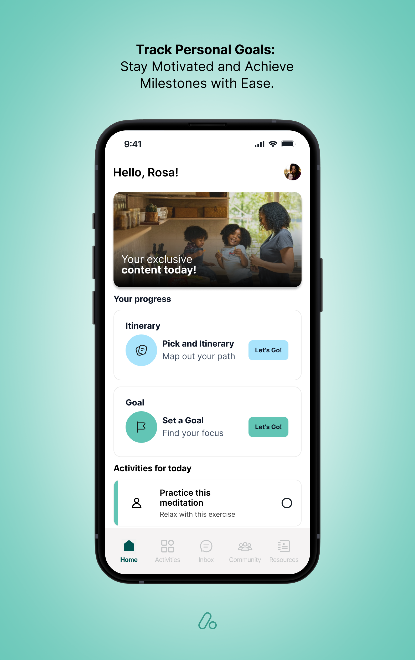

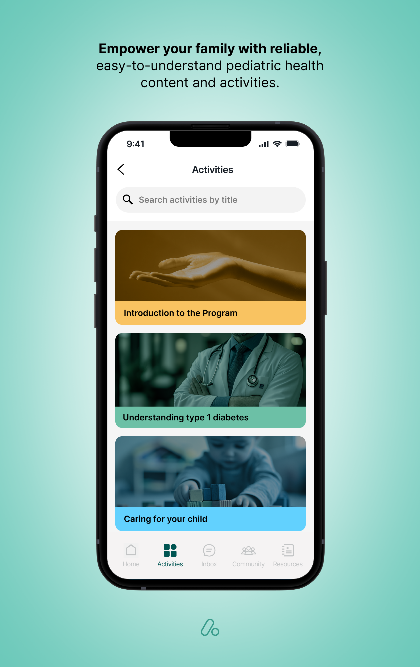

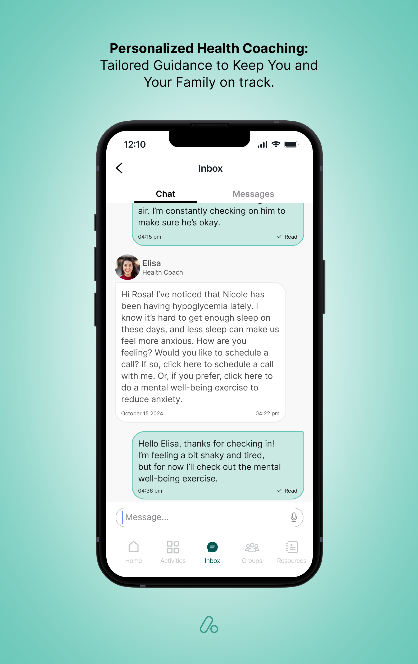

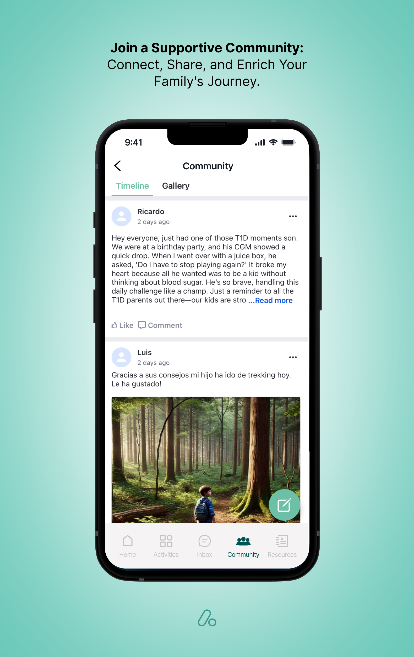

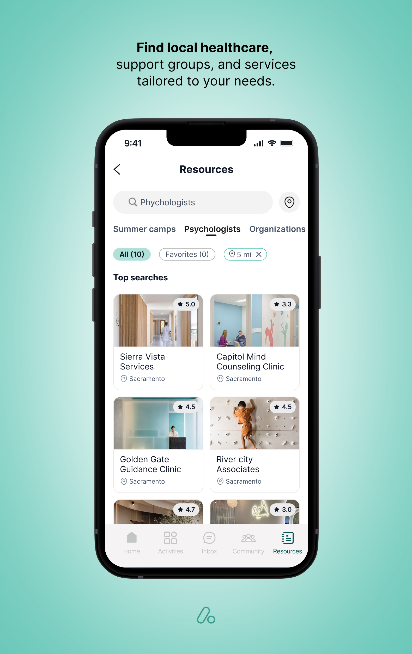

Supplement: Multimedia Appendix 1 [file pediatrics-v8-e66914-s001.docx]
